# Can data from disparate long-term fish monitoring programs be used to increase our understanding of regional and continental trends in large river assemblages?

**DOI:** 10.1371/journal.pone.0191472

**Published:** 2018-01-24

**Authors:** Timothy D. Counihan, Ian R. Waite, Andrew F. Casper, David L. Ward, Jennifer S. Sauer, Elise R. Irwin, Colin G. Chapman, Brian S. Ickes, Craig P. Paukert, John J. Kosovich, Jennifer M. Bayer

**Affiliations:** 1 U.S. Geological Survey, Western Fisheries Research Center, Columbia River Research Laboratory, Cook, Washington, United States of America; 2 U.S. Geological Survey, Oregon Water Science Center, Portland, Oregon, United States of America; 3 Illinois Natural History Survey, Illinois River Biological Station, Havana, Illinois, United States of America; 4 U.S. Geological Survey, Southwest Biological Science Center, Grand Canyon Monitoring and Research Center, Flagstaff, Arizona, United States of America; 5 U.S. Geological Survey, Upper Midwest Environmental Sciences Center, La Crosse, Wisconsin, United States of America; 6 U.S. Geological Survey, Alabama Cooperative Fish and Wildlife Research Unit, School of Forestry and Wildlife Sciences, Auburn University, Auburn, Alabama, United States of America; 7 Oregon Department of Fish and Wildlife, Ocean Salmon and Columbia River Program, Clackamas, Oregon, United States of America; 8 U.S. Geological Survey, Missouri Cooperative Fish and Wildlife Research Unit, The School of Natural Resources, University of Missouri, Columbia, Missouri, United States of America; 9 U.S. Geological Survey, Core Science Analytics, Synthesis, & Libraries, Lakewood, Colorado, United States of America; 10 U.S. Geological Survey, Northwest Region & Pacific Northwest Aquatic Monitoring Partnership, Seattle, Washington, United States of America; Aberystwyth University, UNITED KINGDOM

## Abstract

Understanding trends in the diverse resources provided by large rivers will help balance tradeoffs among stakeholders and inform strategies to mitigate the effects of landscape scale stressors such as climate change and invasive species. Absent a cohesive coordinated effort to assess trends in important large river resources, a logical starting point is to assess our ability to draw inferences from existing efforts. In this paper, we use a common analytical framework to analyze data from five disparate fish monitoring programs to better understand the nature of spatial and temporal trends in large river fish assemblages. We evaluated data from programs that monitor fishes in the Colorado, Columbia, Illinois, Mississippi, and Tallapoosa rivers using non-metric dimensional scaling ordinations and associated tests to evaluate trends in fish assemblage structure and native fish biodiversity. Our results indicate that fish assemblages exhibited significant spatial and temporal trends in all five of the rivers. We also document native species diversity trends that were variable within and between rivers and generally more evident in rivers with higher species richness and programs of longer duration. We discuss shared and basin-specific landscape level stressors. Having a basic understanding of the nature and extent of trends in fish assemblages is a necessary first step towards understanding factors affecting biodiversity and fisheries in large rivers.

## Introduction

Navigable or non-wadeable rivers (hereafter referred to as large rivers), provide valuable resources to a range of socio-economic sectors including: biodiversity, culturally and economically valuable fisheries [1,2], electricity [3], municipal drinking water [4], and water for agricultural and industrial businesses [5]. Consequently, there is a diverse array of stakeholders with a vested interest in the services derived from large rivers. Understanding the status and trends of biological, chemical, economic (e.g., benefits from recreation, commercial and recreational fisheries), and physical (e.g., water availability, habitats) resources will inform management decisions and help stakeholders reach consensus on strategies to mitigate the effects of landscape scale stressors. Long-term resource monitoring data can help provide information that assesses the response of large river resources to management practices. Multiple basin-specific long-term large river monitoring efforts are being conducted in the U.S. [6,7,8]; however, there is currently little coordination across basins and assessments of regional and national trends in large river natural resources are lacking.

Large river monitoring programs are designed for the specific conditions and stakeholder information needs within a particular river system; consequently, these programs often have different goals, designs, and methods. Although the resulting system-specific programs are tailored to individual rivers and issues, these programs typically collect a subset of parameters that are shared across all basins (e.g., abundance of fish species over time, georeferenced habitat characterizations, water temperature). However, few comparisons of trends in resources measured across large river systems have been done. Monitoring programs that allow regional and national assessments of resources in various aquatic systems have been developed. For example, the U.S. Environmental Protection Agency’s (USEPA) Environmental Monitoring and Assessment Program (EMAP) developed standardized methods that were used to monitor and assess the status and trends of national ecological resources from 1990–2006 [9]. While EMAP had a component that dealt specifically with large river fish assemblages, data collection for this component was initiated towards the end of the program. More recently, EMAP has transitioned into the National Aquatic Resource Surveys (NARS) that provides regional and national assessments of trends in aquatic resources; including sites located in large river systems [10]. Similarly, the U.S. Geological Survey’s (USGS) National Water-Quality Assessment Program (NAWQA) provides national assessments of the ecological health of streams and also encompasses some large river systems [11]. While NARS and NAWQA facilitate comparisons of ecological conditions at the landscape/continental scale, neither is intended to specifically assess the status and trends of aquatic resources in large rivers.

Fish assemblages integrate the effects of degradation at all levels and are good indicators of aquatic ecosystem health [12]. For example, the environmental requirements of fish integrate many attributes of physical habitat, water quality, environmental contamination, habitat fragmentation, and overall ecosystem productivity [10,11,12,13]. Since fish rely on multiple trophic levels within an ecosystem throughout their life history they are also affected by the degradation of plant and animal communities they interact with. In addition, many fish populations are actively managed, are long-lived, and can move long distances to take advantage of food resources or life stage specific habitat needs such as spawning or rearing. Fishes also provide many economic benefits to businesses that serve recreational interests, commercial and recreational fishers, tribal members for whom fish are an integral part of their cultural identity [2], and to local and state governments who derive revenue from these activities. Consequently, the status and trends of fishes are of interest to multiple stakeholder groups across multiple jurisdictions.

Having a basic understanding of trends in fish assemblages is necessary to understand the effects of landscape level stressors such as climate change, hydropower development, invasive species, urbanization, and water quality on biodiversity and fisheries in large rivers. Absent a cohesive coordinated effort to assess trends in large river fish assemblages, a logical starting point is to assess our ability to draw inferences from existing monitoring efforts. We began by identifying and analyzing data from programs that monitor fishes in several large rivers across the United States. The data from these programs have been used to help understand the movement and population abundance of fishes listed under the Endangered Species Act (ESA) [8], fish population responses to management actions [14,15], changes in regulation and policy [7], arrival of invasive species [16], and to assess biological integrity [17]. In this paper, we assess whether we can use data from five disparate fish monitoring programs in the Colorado, Columbia, Illinois, Mississippi, and Tallapoosa rivers to better understand the nature of spatial and temporal trends in large river fish assemblages. If we can discern spatial and temporal trends in fish assemblages, these data could then be used to evaluate hypotheses about the effects of management actions and landscape level stressors on large river fishes.

## Materials and methods

### Monitoring programs

**Fig 1:**
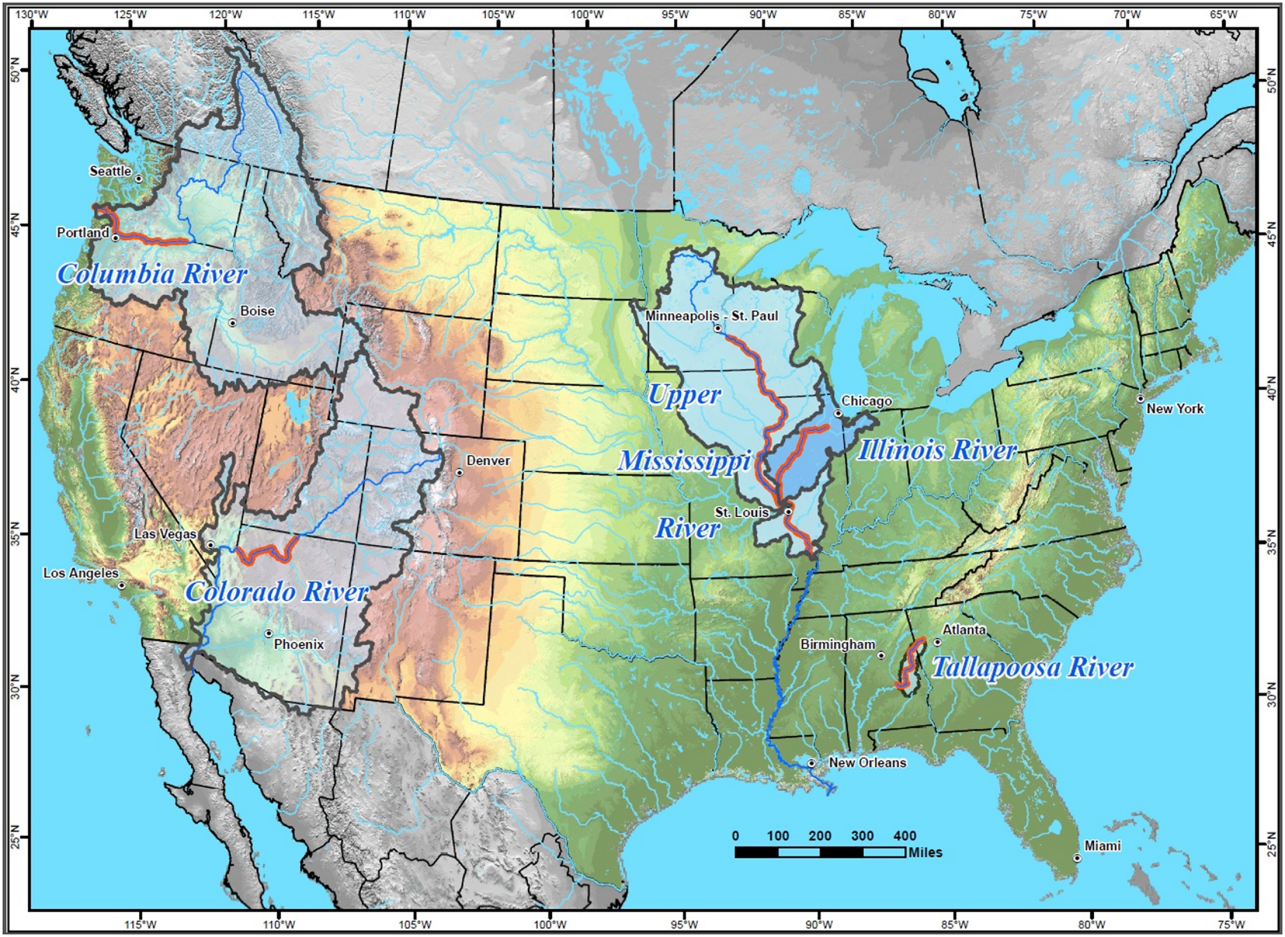
Map of the rivers in which the fish monitoring programs evaluated were conducted. The rivers are the Colorado River in Arizona, Columbia River along the borders of Oregon and Washington, Illinois River in Illinois, Mississippi River along the borders of Illinois, Iowa, Minnesota, Missouri and Wisconsin, and the Tallapoosa River in Alabama. River reaches evaluated in this study are highlighted in red.

Monitoring programs from the Colorado, Columbia, Illinois, Mississippi, and Tallapoosa rivers provided the basis for our analyses (Fig 1). The design, duration, field collection methods, intended purposes, and authority for the conduct of the programs differ among rivers [18].

#### Colorado River

The Colorado River flows 2,330 km from its origin in Colorado to its confluence with the Gulf of California in Mexico draining parts of seven U.S. and two Mexican states; our assessment focused on the portion of the Colorado River that flows through the Grand Canyon in Arizona. The Colorado River is heavily developed to provide water for agriculture, municipalities, and electricity. The focus of the fish monitoring within the Colorado River is the conservation of fishes listed under the ESA [18]. Monitoring data collected by the USGS Grand Canyon Monitoring and Research Center and its various cooperators since 1996 have documented improvements to several key biotic and abiotic resources leading to improved understanding of the ecosystem.

We summarized data collected in three reaches of the Colorado River that begin below Glen Canyon Dam and traverse through the Grand Canyon to near where the Colorado River becomes Lake Mead. The Upper reach extends from river kilometer (rkm) 0 (i.e., Lee's Ferry which is at the confluence of the Paria River 26 rkm downstream from the Glen Canyon Dam base)-rkm 99 (the confluence of the Colorado River with the Little Colorado River); the Middle reach extends from rkm 99–290 (Lava Falls); and the Lower reach extends from rkm 290-447(Lake Mead).

#### Columbia River

The Columbia River is the fourth largest river by volume in the United States [19] and is home to iconic anadromous fish species such as Chinook (*Oncorhynchus tshawytscha*), coho (*O*. *kisutch*), chum (*O*. *keta*), and sockeye salmon (*O*. *nerka*); including several populations which are listed as threatened or endangered under the ESA. The Columbia River originates in British Columbia, Canada, has a basin area of 668,217 km^2^, and flows 2010 km to its confluence with the Pacific Ocean. The Columbia River monitoring effort is designed to provide a reach-specific annual index of abundance for age-0 white sturgeon (*Acipenser transmontanus*) [18].

The reaches for the Columbia River are delineated by hydroelectric dams. The four reaches evaluated in this study correspond to John Day, The Dalles, and Bonneville reservoirs, the three lowest most impoundments on the Columbia River, and the Columbia River Estuary. The upriver most reach is the John Day reach which is impounded by John Day Dam (rkm 348) on the lower end and extends to McNary Dam (rkm 469) on the upriver end. The Dalles reach is the next reach downriver and is impounded by The Dalles Dam (rkm 309) on the lower end and extends to John Day Dam on the upriver end. The Bonneville reach, which traverses the Cascade Mountains, is impounded by Bonneville Dam (rkm 235) on the lower end and extends to The Dalles Dam on the upriver end. The Estuary reach is the lowermost reach and extends from below Bonneville Dam to the confluence with the Pacific Ocean.

#### Illinois River

The Illinois River is 439 km long and has a basin area of 75,136 km^3^. Geomorphologically, the river can be split into an agriculturally dominated lower portion that is turbid, alluvial with a wide floodplain and numerous backwater habitats, which contrasts with the upper portion that flows out of an urbanized and glacially dominated region, augmented by oligotrophic Lake Michigan base discharge [20,21,22]. With 5 navigation dams, an artificially maintained 3 m navigation channel, the Chicago metro area in its headwaters, and with approximately 50% of the floodplain behind levees in the lower portion of the river, the Illinois River is a heavily modified ‘working’ river [23]. Despite these constraints the Illinois River still supports approximately 127 fish species that provide both commercial and recreational fisheries [20]. The monitoring program for the Illinois River was initiated in 1957. Initially the program was focused on documenting the effects of pollution on the river fish assemblage however as water quality has improved dramatically since the 1970’s this focus has shifted to documenting the status and trends of recreational and commercially harvested species [7,24]. More recently the arrival of invasive species, especially bighead carp *Hypophthalmichthys nobilis* and silver carp *Hypophthalmichthys molitrix* has resulted in changes in both fish condition factors and the fish assemblage [16,25].

The data from the Illinois River comes from a monitoring effort at 27 fixed main channel border sites that are spread across six reaches. The Dresden reach on the Des Plaines River ranges from rkm 0–21 above Marseilles reach. The Marseilles reach ranges from rkm 393-435km (rkm 435 = source of Illinois River). The Starved Rock reach ranges from rkm 367 to 393. The Peoria reach ranges from rkm 250–367. The LaGrange reach ranges from rkm 126–250, and the Alton reach that ranges from rkm 0 (confluence with Mississippi)-rkm 126; the five reaches encompass the Illinois River near its origin near Chicago, IL to its confluence with the Mississippi River [7].

#### Upper Mississippi River

The Upper Mississippi River (UMR) is a 2000 km long reach of the Mississippi River upstream of Cairo, Illinois. The ecosystem includes a wide array of fish and wildlife species distributed across a complex assortment of flowing channels, floodplain lakes, backwaters, wetlands, and floodplain forests. Uses of the UMR range from commercial navigation and water supply to supporting outdoor recreation and fish and wildlife habitat. The focus of the U.S. Army Corps of Engineers Upper Mississippi River Restoration Program’s Long Term Resource Monitoring (LTRM) element is to assess the ecological response to navigation, navigation infrastructure, and habitat rehabilitation [18]. The LTRM has transitioned from its initial phase of developing a data collection system and ecological database to a second phase of using those data for increased understanding of river process and function, developing indicators of ecosystem health, and evaluating management success. The LTRM is implemented by the USGS in partnership with the US Army Corps of Engineers, and multiple state agencies.

The reaches evaluated as part of these analyses are Pools 4, 8, 13 and 26 and an un-impounded reach that extends downriver from Pool 26 to near the confluence of the Ohio River. In contrast to the reaches in the Columbia, Colorado, and Illinois rivers, the reaches evaluated for the Mississippi River are not contiguous. Given that rkm 0 occurs at the confluence of Ohio and Mississippi rivers near Cairo, IL, Pool 4 is the upriver most reach and extends from rkm 1210–1282; Pool 8 is the next reach downriver and extends from rkm 1093–1131; Pool 13 extends from rkm 840–896; Pool 26 extends from rkm 322–389; and the Open River reach extends from 1–130km.

#### Tallapoosa River

The Tallapoosa River has its headwaters in the Georgia piedmont and flows 426 km where it meets the Coosa River to form the Alabama River. The river has four privately owned hydropower dams along its course; the upper most dam, R.L. Harris Dam, is the most recently installed and closed in 1983. The fauna is diverse and includes 57 fish species including seven endemic species [26]. Shortly after the R.L. Harris Dam closed, negative information regarding the discharge regime and its perceived impacts on fisheries and biodiversity began to be reported to Federal and State conservation managers [27]. In 2003, a stakeholder workshop initiated the development of a decision model to assist in determining a starting point for measuring the response of multiple objectives to changes in the discharge regime [28]. Maintaining productive fishery resources was a fundamental objective of several stakeholder groups and fish population responses to management became one of the primary measures of management success. In order to determine if fishery resource objectives were being met by the river discharge delivered, long-term monitoring of fishes was implemented in 2005 as part of an ongoing and formal adaptive management program [29,30].

The reaches on the Tallapoosa River evaluated in this study consist of areas above and below R.L. Harris Dam. The Upper reach is located above R.L. Harris Dam and extends from rkm 266–271. The Malone reach is located downriver of the dam and extends from rkm 193–201. The Wadley reach ranges from rkm 184–192. The Lower reach ranges from rkm 135–145.

#### Field methods

Since electrofishing was used to collect fish in all but one of the rivers evaluated (i.e., Columbia River), we compiled data from electrofishing efforts for most of the monitoring programs to estimate the abundance of fish species. For the Colorado River, fish were collected as part of fish assemblage monitoring that has been conducted annually since 1991 from Glen Canyon Dam to the Lake Mead inflow (482 km) using boat-mounted, DC electrofishing conducted at night. Sampling design methods were standardized in 2000 and now utilize a stratified random sampling design to estimate a catch-per-unit-effort index for each fish species encountered [31,32]. Fish collection efforts conducted from March through June from 2001–2013 were used.

For the Columbia River, fish were collected using small-mesh gill nets fished in the fall (October- November) of each year at fixed locations within each reach. Gill-nets measured 91.4-m in length and 3.7 m in height and were constructed with 5.1cm stretched-measure multifilament nylon webbing. Nets were fished on the river bottom overnight for approximately 24 hours. At the end of each 24-hour sampling period, the nets were retrieved and redeployed in the same location for the next 24-hour sampling period. Data were available for John Day and The Dalles reaches from 1997–2013, for Bonneville reach from 2006–2013, and for the Estuary reach from 2004–2006 and from 2011–2013. Since the gear used was fished on the river bottom, the sampling targets fish occupying demersal habitats in the Columbia River in contrast to other monitoring programs that used boat electrofishing.

For the Illinois River, we used data from AC boat electrofishing efforts at fixed main channel border sites within the Alton, LaGrange, Peoria, Starved Rock, Marseilles, and Dresden reaches from sampling conducted since 1957 in most reaches [7]. Not all reaches had data as far back as 1957 and some reaches were not sampled in all years. However, sampling has generally occurred annually in most reaches from August-October when water temperatures were above 14°C and the more stable hydrographic conditions in summer and early-fall are established.

For the UMR, we used data from electrofishing that has been conducted annually since 1993 from June through November with pulsed direct current using boat configurations and power outputs that are standardized [33]. Electrofishing effort is of 15-min duration and is paced so that the boat covers a rectangle of about 200 × 30 m; the unit of effort is a 15-min run. In the UMR, sites are selected using a stratified random sampling scheme within reaches.

For the Tallapoosa River, sampling is conducted using pre-positioned area electrofishers (PAEs) to sample fixed sites within each reach that are allocated to shoal habitats [34]. PAEs (6 x 1.5 m; n = 10–20) are set in shallow habitats (< 0.75 m), left undisturbed for 20 minutes, and powered remotely using a Smith Root 2.5 GPP using alternating current. A seine is set downstream of the PAE and workers visually inspect and systematically remove stunned fish and then kick through the PAE to dislodge fish that may be impinged behind substrata or vegetation. Samples have been collected annually from May-November since 2005 [35].

### Data analyses

For each river, data were obtained as the number of each fish species captured by sampling location and year. Prior to compiling the data, the principal investigator for each monitoring program worked with a GIS analyst to identify the full extent of the river system that was sampled and how sampling locations on the river should be grouped to create the river reaches used in the analyses. For the Columbia, Mississippi, and Illinois Rivers, the geographic extent of reaches was primarily based on the presence of man-made structures (e.g. Dams). In the Colorado and Tallapoosa Rivers, reaches were based on expert judgement of the locations of changes in river habitat conditions. A master species list was compiled for all five rivers. We standardized fish species codes in the master species list across programs and referenced scientific and common names for each species. In addition, the native status of each species for each of the five rivers was determined and included in the master species list.

To characterize the abundance of fishes, we estimated the mean number of fish per species captured per effort (e.g., electrofishing grid, electrofishing run, or gill net set) by reach and year for each of the rivers. We then used the mean number of fish per species captured per effort by reach and year to perform a non-metric dimensional scaling ordination (NMDS) on a Bray-Curtis similarity matrix for each river. The estimated abundance values were log transformed (log (x+1)). The ordination was performed in Primer-e [36]. Analysis of Similarity tests (ANOSIM) were used to test the significance of observed differences between the monitoring data longitudinally within rivers (i.e., by reach) and between specific time periods for each river. The product of ANOSIM is a *p*-value and a Global R value that ranges from −1 to +1, with values of 0 indicating the null hypothesis (no difference among groups) and values closest to 1 indicating that groups differ in assemblage composition. We used post-hoc Similarity of Percentages (SIMPER) to identify the species primarily contributing the most to differences detected between groups. The time periods evaluated for each river were selected based on expert judgement with the primary purpose of providing temporal context to the data. Time periods were coarsely delineated by decade for rivers with longer time series (e.g., Illinois River) or finer time scales for rivers with shorter time series (e.g., Tallapoosa River).

For each river we then used the average catch of native fishes by reach and year to generate k-dominance plots for native fishes by reach and for two years that span the duration of a particular monitoring program. The k-dominance plots relate the cumulative relative abundance (i.e. percentage of the total abundance in a sample) to cumulative rank [36]. Species evenness and dominance are reciprocal assemblage characteristics that help us better understand and characterize species richness and diversity. In the comparison of a disturbed or degraded ecosystem with a less disturbed ecosystem, both frequently have a similar number of species but in the disturbed ecosystem, the relative abundances are not usually as even as in an undisturbed ecosystem [36].

### Environmental and human population data

To provide context to the fish assemblage data in each of the river basins, we compiled human population data for relevant Metropolitan Statistical Areas (MSA), data describing environmental conditions in each river, and data that describe important management and regulatory events. Population data for the MSA in each river basin were derived from US Census Bureau data and presented as 10-year moving averages. For the Colorado River, we compiled water temperature data collected by the USGS at Lees Ferry (Upper reach; USGS gage 09380000). Using these data, we estimated 10-year moving averages of the mean daily water temperature during May-July to illustrate trends in water temperature arising from land and river management and climate. For the Columbia River, we estimated 10-year moving averages of the proportion of annual river discharge occurring in June from river discharge data collected by the US Army Corps of Engineers to illustrate the effects of land and river management and climate on the seasonality of discharge. We also compiled river discharge for the Illinois River near Havana, Illinois, the Mississippi River near St. Louis, Missouri, and the Tallapoosa River near Wadley, Alabama from data collected by the USGS. For the Illinois, Mississippi, and Tallapoosa Rivers we estimated the mean annual river discharge and present the data as 10-year moving averages to characterize the effects of land and river management and climate on trends in annual river discharge.

## Results

The ordination of the fish abundance data revealed spatial and temporal patterns suggesting that the fish assemblages in all these rivers are in a state of flux. A total of 215 species of fish, excluding hybrids, were collected by the monitoring programs evaluated (S1 Table). The river system with the greatest number of native fish species captured was the UMR (*n* = 138), while the Colorado River had the lowest number of native species captured (*n* = 5). The percentage of fish assemblage comprised of introduced species ranged from 85% in the Colorado River to 2% in the Tallapoosa River.

**Fig 2:**
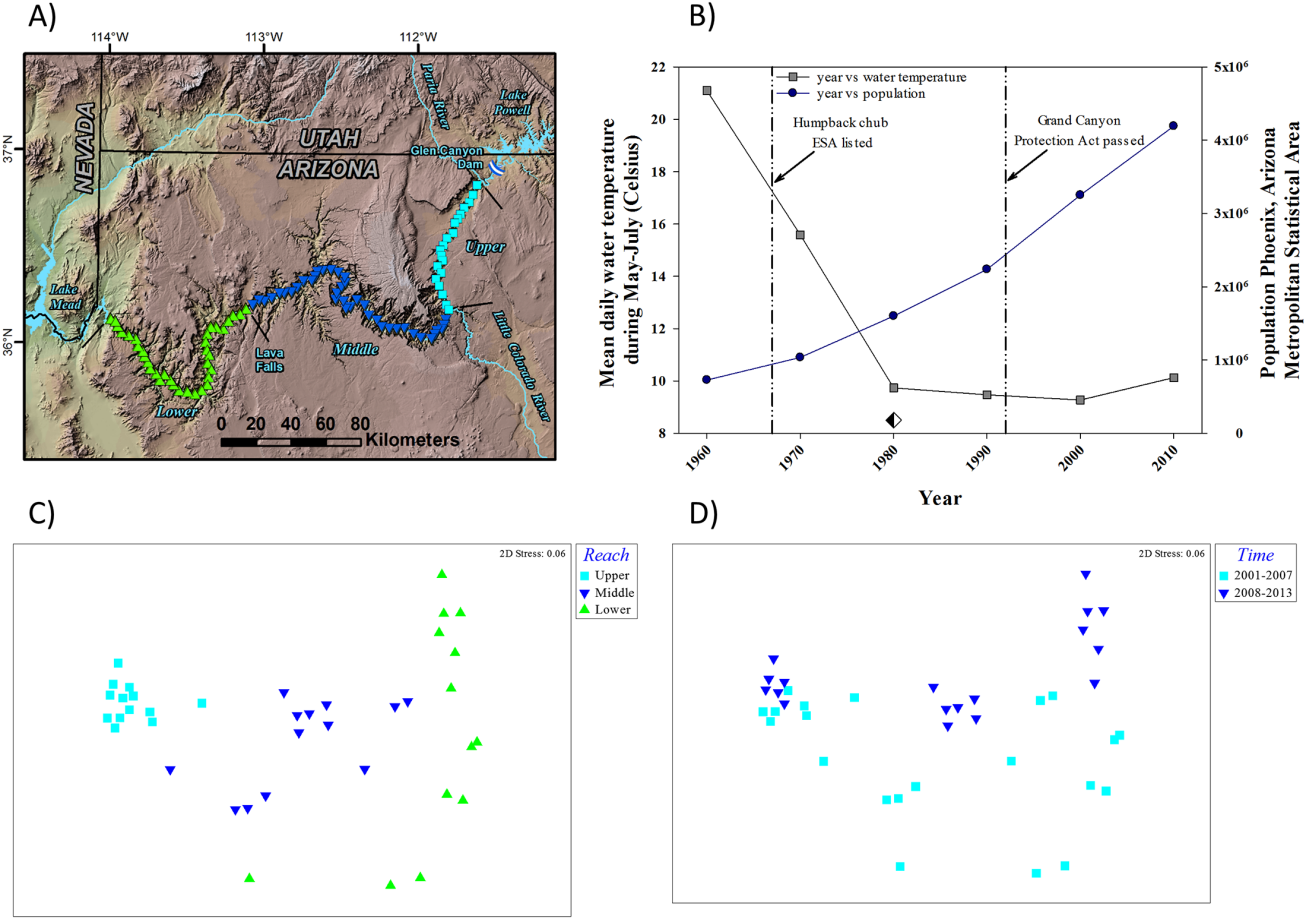
A) A map of study reaches specifically covered in the Colorado River. Electrofishing locations are designated by colored symbols that correspond to shapes and colors in the non-metric multidimensional scaling ordination (NMDS) of mean species abundances by reach in panel C) described below. B) Temporal trends in the population of the Phoenix, Arizona Metropolitan Statistical Area (blue circles) and in the mean daily water temperature (Celsius) in the Upper reach during May-July (gray squares). Both the population and temperature data are presented as 10-year moving averages. The black and white diamond indicates the year Lake Powell was filled. The vertical reference lines denote important regulatory milestones. C) A NMDS of mean species abundances by reach as derived from electrofishing efforts conducted annually in the Colorado River from 2001–2013. D) The NMDS ordination coded by time period (2001–2007 and 2008–2013).

For the Colorado River ordination, we observed three groupings that correspond to the three river reaches examined (Fig 2). The ANOSIM indicated significant differences in fish assemblages between the three reaches (*p* = 0.001; *Global R* = 0.776) and for all pairwise comparisons (*p* = 0.001). The SIMPER results suggested that fish assemblage differences resulted from shifts in abundances of rainbow trout *Oncorhynchus mykiss*, flannelmouth sucker *Catostomus latipinnis*, brown trout *Salmo trutta*, and speckled dace *Rhinichthys osculus* between reaches. In addition to the spatial trends we also observed temporal trends. When the data were aggregated into collections occurring from 2001–2007 and 2008–2013, ANOSIM indicated significant differences between the time periods for the upper (*p* = 0.007; *Global R* = 0.37), middle (*p* = 0.048; *Global R* = 0.24), and lower (*p* = 0.001; *Global R* = 0.72) reaches (Fig 2D). Analysis of Similarity of the temporal trends indicated that species responsible for differences between the two time periods were rainbow trout, flannelmouth sucker, brown trout, and speckled dace for the upper reach; between rainbow trout, flannelmouth sucker, brown trout, common carp *Cyprinus carpio*, red shiner *Cyprinella lutrensis*, and bluehead sucker *Catostomus discobolus* for the middle reach, and between flannelmouth sucker, common carp, bluehead sucker, and red shiner for the lower reach.

**Fig 3:**
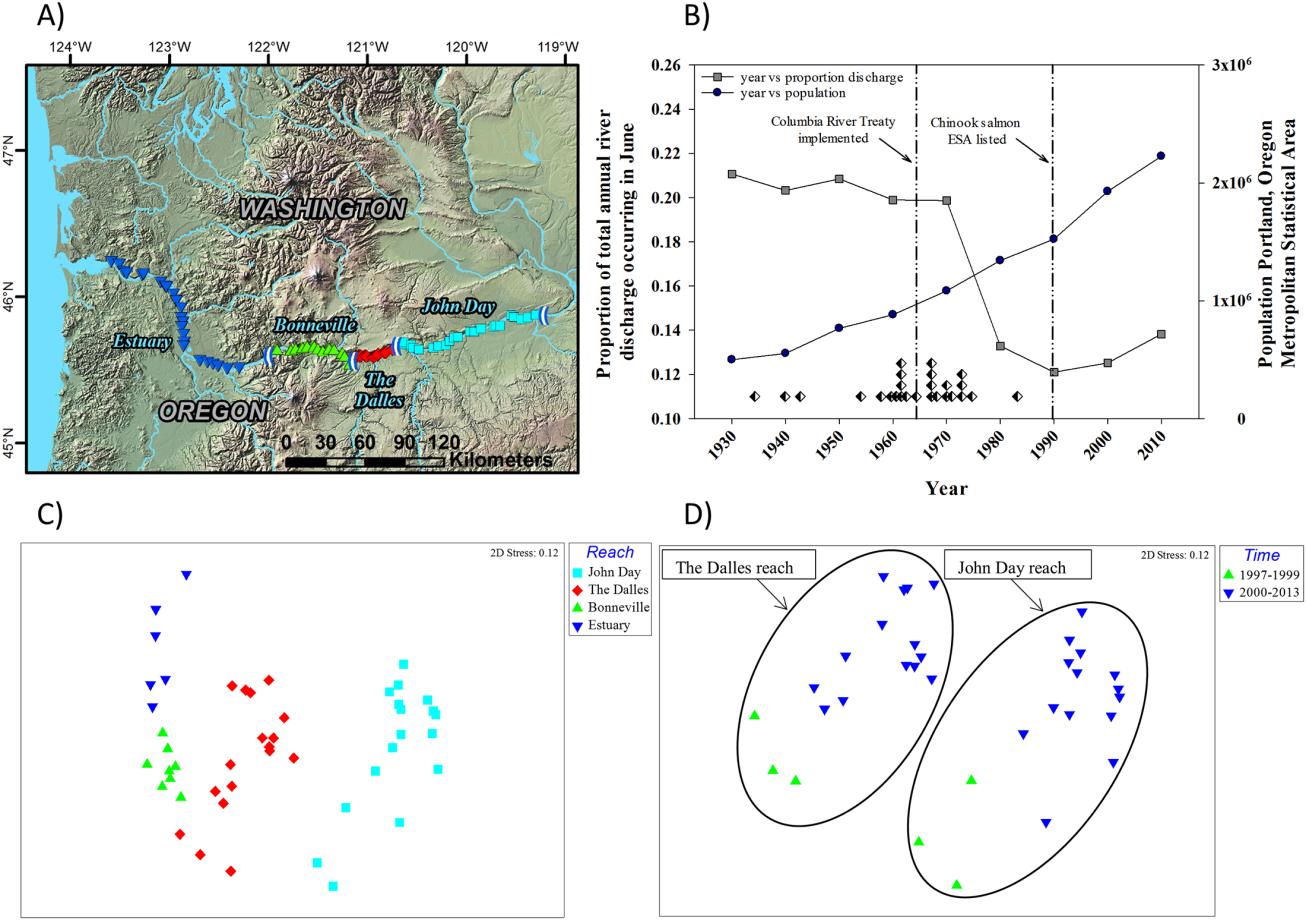
A) A map of study reaches specifically covered in the Columbia River. Gill net sampling locations are designated by colored symbols that correspond to shapes and colors in the non-metric multidimensional scaling ordination (NMDS) of mean species abundances by reach in panel C) described below. B) Temporal trends in the population of the Portland, Oregon Metropolitan Statistical Area (blue circles) and in the proportion of total annual discharge occurring in June at The Dalles Dam (gray squares). Both the population data and proportion of total annual discharge occurring in June are presented as 10-year moving averages. The black and white diamonds represent major dam completion events in the Columbia River Basin. The vertical reference lines denote important regulatory milestones. C) A NMDS of mean species abundances by reach as derived from small-mesh gill net efforts conducted annually in the Columbia River from 1997–2013 in John Day and The Dalles reaches, from 2006–2013 in the Bonneville reach, and from 2004–2006 and 2011–2013 in the Estuary reach. D) A NMDS that includes only John Day and The Dalles reaches coded by time period (1997–1999 and 2000–2013).

For the ordination of the Columbia River data, we observed four groupings that corresponded to the four reaches in the analyses (Fig 3). The ANOSIM results indicated significant differences between reaches (*p* = 0.001; *Global R* = 0.728) and for all pairwise comparison (*p* < 0.005). The SIMPER analyses indicated some consistency in the nature of the differences in abundances of fish species between reaches. For instance, the dissimilarities between reaches were typically a result of differences in the abundances of native fishes such as the peamouth chub *Mylocheilus caurinus* and white sturgeon *Acipenser transmontanus* and non-native fishes such as yellow perch *Perca flavescens*, channel catfish *Ictalurus punctatus*, and walleye *Sander vitreus*. In general, the SIMPER analyses indicated that non-native fishes became less abundant from upriver to downriver. For the reaches where the time series was long enough to examine temporal trends (i.e., John Day and The Dalles reaches) temporal trends in fish assemblage structure were observed. When the data were grouped into samples taken from 1997–1999 and from 2000–2013, the results of the ANOSIM indicated significant differences in fish assemblage structure in John Day (*p* = 0.001; *Global R* = 0.873) and The Dalles (*p* = 0.001; *Global R* = 0.799) reaches (Fig 3D). For both The Dalles and John Day reaches, the SIMPER results suggested a higher abundance of non-native yellow perch and a lower abundance of native white sturgeon and largescale sucker *Catostomus macrocheilus* during 200–2013; largescale sucker, white sturgeon, and yellow perch each contributed > 10% to the dissimilarity between time periods.

**Fig 4:**
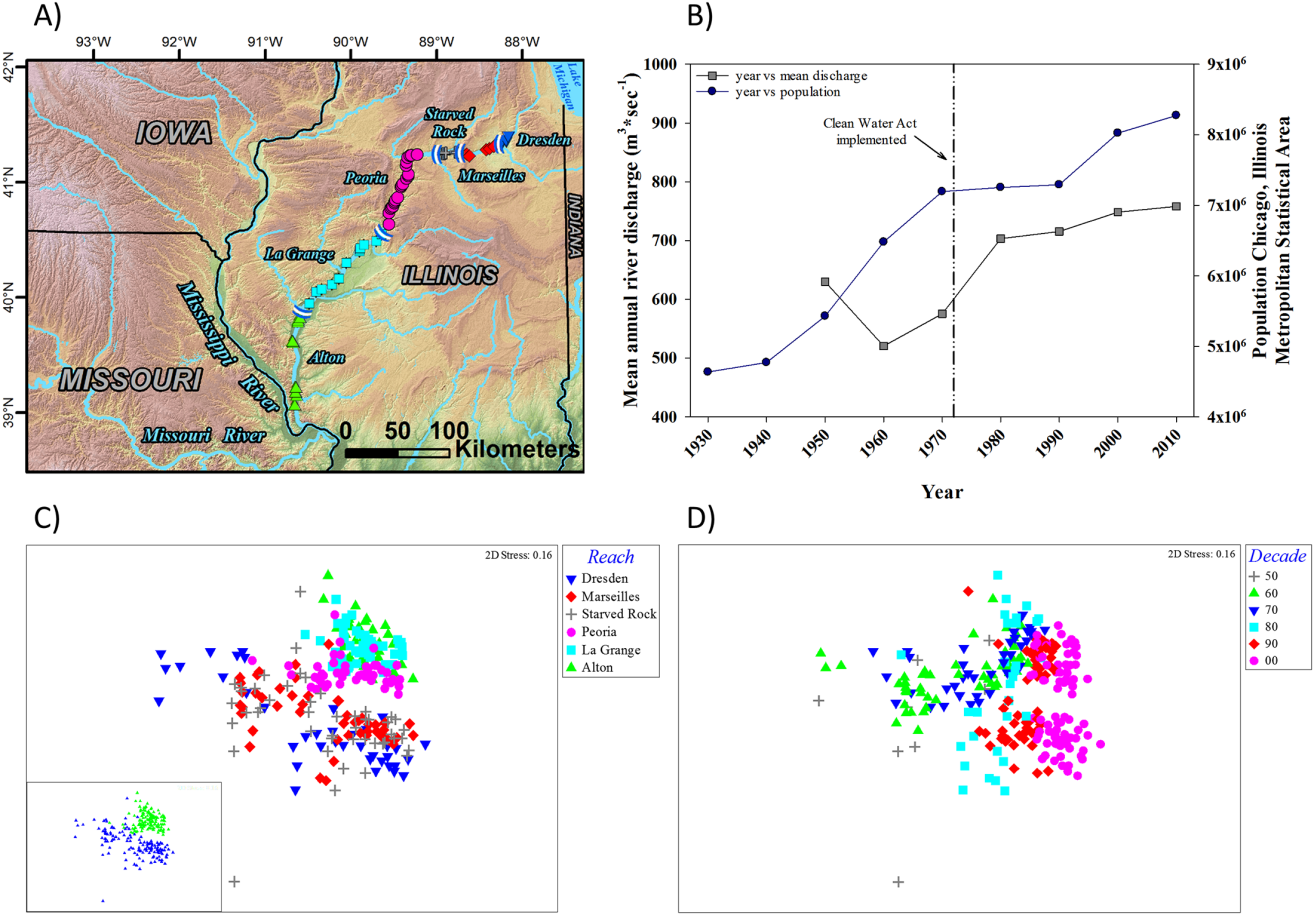
A) A map of study reaches specifically covered in the Illinois River. Electrofishing locations are designated by colored symbols that correspond to shapes and colors in the non-metric multidimensional scaling ordination (NMDS) of mean species abundances by reach in panel C) described below. B) Temporal trends in the population of the Chicago, Illinois Metropolitan Statistical Area (blue circles) and in the mean annual river discharge (m ^3^ * sec ^-1^) at near Havana, Illinois (gray squares). Both the population data and mean annual river discharge are presented as 10-year moving averages. The black and white diamonds represent lock and dam completion events in the Illinois River Waterway System. The vertical reference line denotes an important regulatory milestone. C) A NMDS of mean species abundances by reach as derived from electrofishing efforts conducted annually in the Illinois River from 1957–2013. The panel inset shows the NMDS grouped by the Dresden, Marseilles, and Starved Rock reaches (blue triangles) and by the Peoria, La Grange, and Alton reaches (green triangles). D) The NMDS of mean species abundances by reach coded by time period (1957–1959 = 50, 1960–1969 = 60, 1970–1979 = 70, 1980–1989 = 80, 1990–1999 = 90, 2000–2013 = 00).

The ordination of the Illinois River data also revealed reach-based groupings; however, the delineation among reaches was less evident than for the Colorado and Columbia rivers (Fig 4). Two clusters are evident: the three most upriver reaches (e.g., Dresden, Marseilles, and Starved Rock) and the other that clustered the three downriver reaches (e.g., Peoria, LaGrange, and Alton) (Fig 4C). Despite the lack of clear delineation of groups at the reach level in the NMDS plot, the results of the ANOSIM indicated significant differences in fish assemblage structure between reaches (*p* = 0.001; *Global R* = 0.43) and for all pairwise reach comparisons (*p* < 0.005). The SIMPER results suggested that the dissimilarities between reaches were a result of small differences in many fish species as opposed to large differences in just a few fish species. The highest percent contribution of one fish species to the total dissimilarity between reaches was 8.1%. The average dissimilarity of the pairwise comparisons ranged from a low of 37.6 between the Alton and LaGrange to high of 64.1 between Dresden and Alton. When we grouped the upper three reaches versus the lower three reaches and performed an ANOSIM, the results confirmed that the fish assemblages were significantly different (*p* = 0.001; *Global R* = 0.508). The SIMPER results show that the dissimilarity is mainly a function of freshwater drum *Aplodinotus grunniens*, emerald shiner *Notropis atherinoides*, bluegill *Lepomis macrochirus*, bluntnose minnow *Pimephales notatus*, gizzard shad *Dorosoma cepedianum*, and bigmouth buffalo *Ictiobus cyprinellus*, each of which contribute > 4% to the dissimilarity between the upper and lower river assemblage. When we grouped the data by decade for NMDS, temporal trends in fish assemblages were evident (Fig 4D). The ANOSIM and all pairwise comparisons results confirm significant differences by decade (*p* = 0.001, *Global R* = 0.376; *p* = 0.001, respectively). The temporal SIMPER results were similar to the upper versus lower spatial comparison in that the significant dissimilarities were the result of multiple fish species; notably the decline of non-native common carp and increase in bluegill through time.

**Fig 5:**
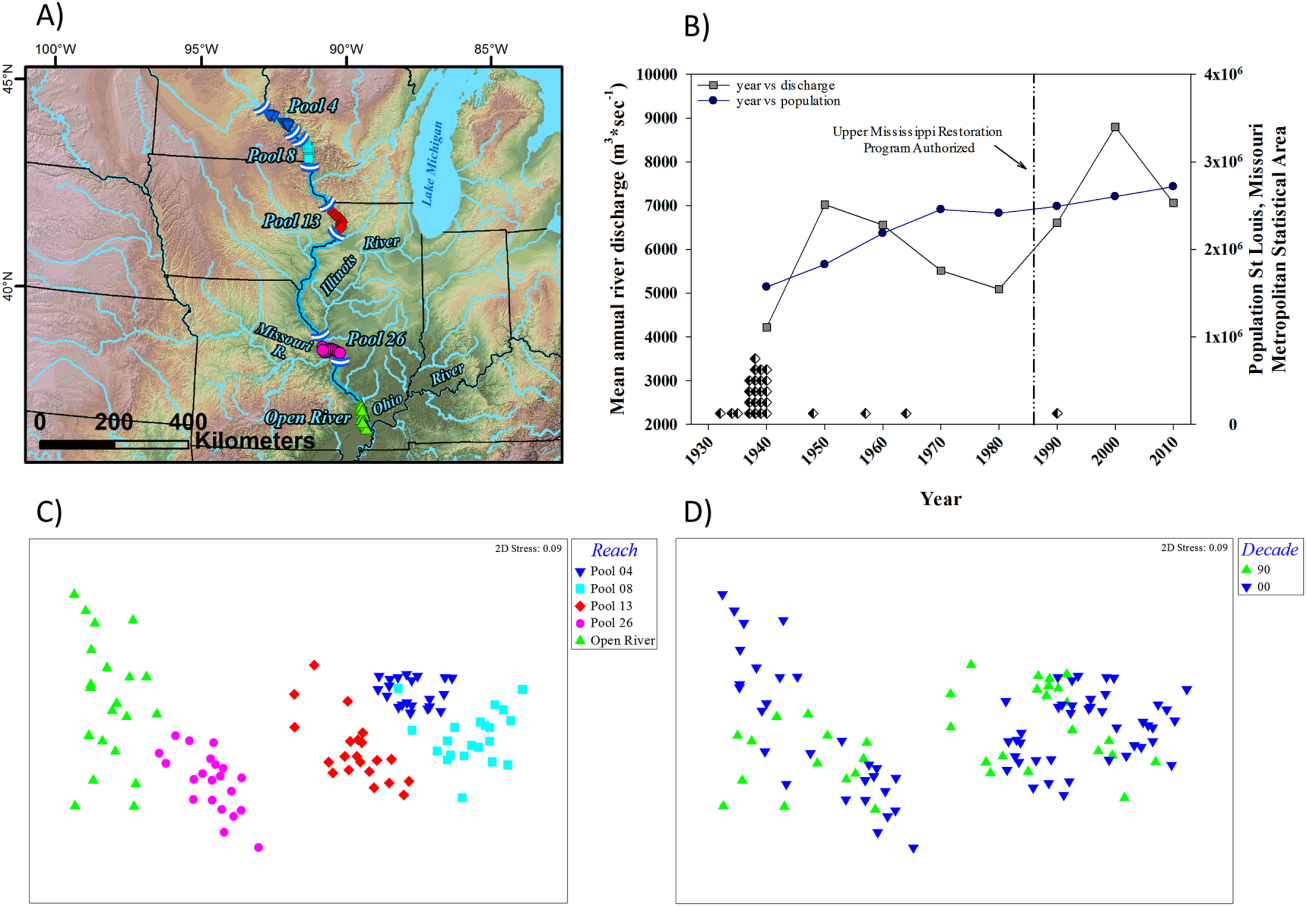
A) A map of study reaches specifically covered in the Upper Mississippi River. Electrofishing locations are designated by colored symbols that correspond to shapes and colors in the non-metric multidimensional scaling ordination (NMDS) of mean species abundances by reach in panel C) described below. B) Temporal trends in the population of the St. Louis, Missouri Metropolitan Statistical Area (blue circles) and in the mean annual river discharge (m ^3^ * sec ^-1^) in July near St. Louis, Missouri (gray squares). Both the population data and mean annual river discharge are presented as 10-year moving averages. The black and white diamonds represent lock and dam completion events in the Upper Mississippi River. The vertical reference line denotes an important regulatory milestone. C) A NMDS of mean species abundances by reach as derived from electrofishing efforts conducted annually in the Mississippi River from 1993–2013. D) The NMDS of mean species abundances by reach coded by time period (1993–1999 and 2000–2013).

Similar to what we observed for the Colorado and Columbia rivers, the ordination of the UMR data revealed groupings that corresponded to the reaches evaluated (Fig 5). The ANOSIM results for the UMR also indicated that there were significant differences between reaches (*p* = 0.001; *Global R* = 0.881) and for all pairwise comparisons (*p* = 0.001). Similar to the Illinois River, the SIMPER results suggested that smaller differences among a large number fish species are contributing to the dissimilarities rather than large differences in a few species as in the Colombia and Colorado rivers. The maximum percent contribution of a single species to the dissimilarity between reaches was 7.3%. The average dissimilarity of the pairwise comparisons ranged from a low of 31.6 between the Pools 4 and 8 (the two most upriver reaches) to 72.4 between the Open River reach and Pool 8 (the downriver most reach and second most upriver reach; respectively). Temporal trends in the data were less evident than for the Columbia, Colorado, and Illinois rivers. However, when we grouped the data by reach into samples collected prior to 2000 (1993–1999) and those occurring after 1999 (2000–2013) (Fig 5D), the results of the ANOSIM show statistically significant differences (Pool 4: *p* = 0.001, *Global R* = 0.473; Pool 8: *p* = 0.001, *Global R = 0*.*542*; Pool 13: *p* = 0.001, *Global R* = 0.513; Pool 26: *p* = 0.001, *Global R* = 0.428; Open River: *p* = 0.007, *Global R* = 0.298). As with the between pool comparison, the SIMPER results contrasting time periods within reaches also suggest that a large number of fish species were contributing to the dissimilarities. Gizzard shad contributed > 4% to the dissimilarities between time periods in all five reaches with increases in average abundance in four of the five reaches. Emerald shiners also contributed > 4% to the dissimilarities between time periods in all five reaches but average abundance decreased in two of the five reaches. Bluegill contributed > 4% to the dissimilarities noted in three upriver reaches (Pools 4, 8, and 13) with increases in average abundance noted in Pools 4 and 8 but not Pool 13. Non-native species did not contribute > 4% in upper most three of the five reaches but the common carp and silver carp *Hypophthalmichthys molitrix*, a recent invader to the UMR, contributed > 4% in Pool 26 and the Open River reach; the two downriver most reaches (Fig 5).

**Fig 6:**
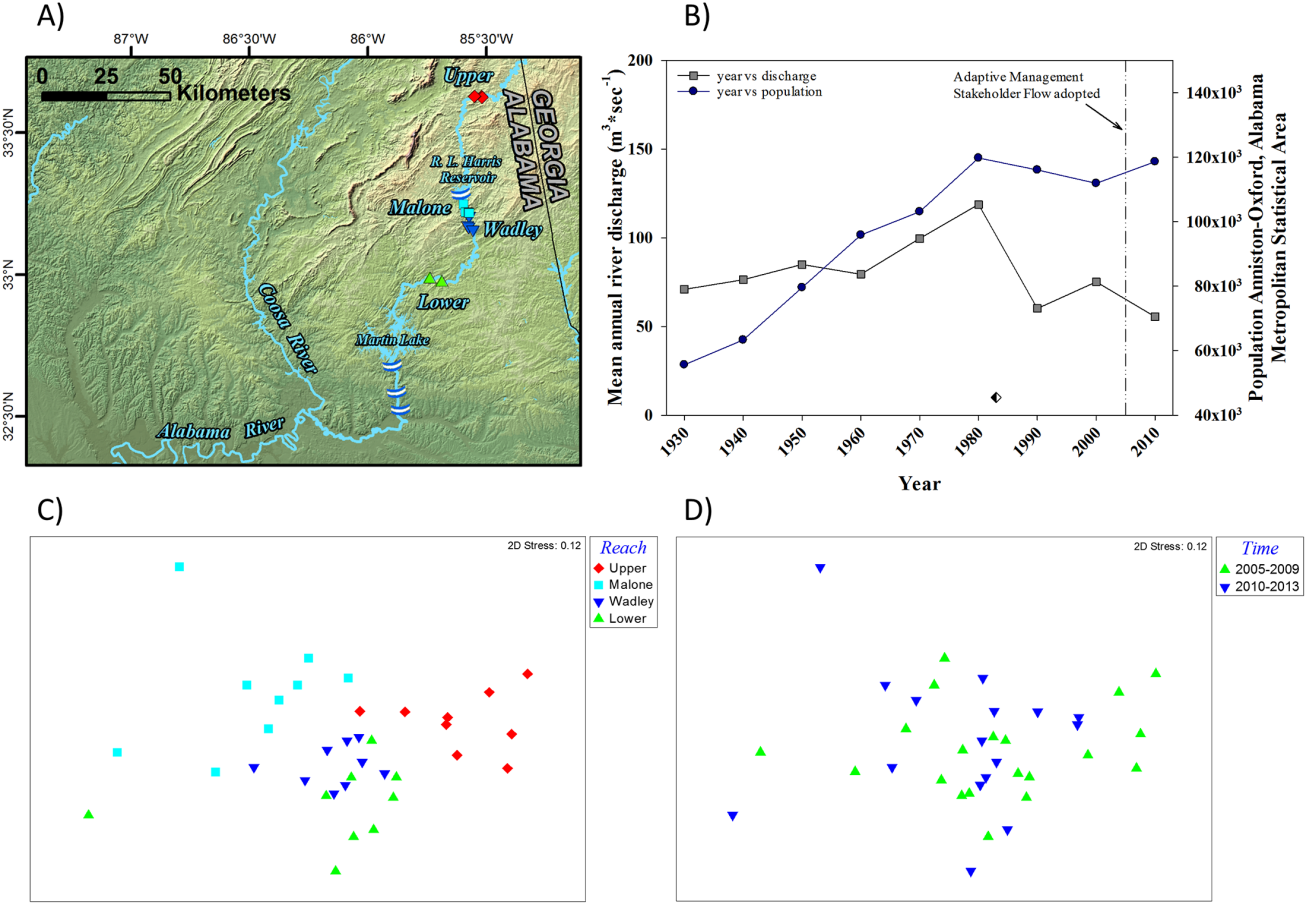
A) A map of study reaches specifically covered in the Tallapoosa River. Electrofishing grid locations are designated by colored symbols that correspond to shapes and colors in the non-metric multidimensional scaling ordination (NMDS) of mean species abundances by reach in panel C) described below. B) Temporal trends in the population in the Anniston-Oxford, Alabama Metropolitan Statistical Area (blue circles) and in the mean annual river discharge (m ^3^ * sec ^-1^) in July near St. Louis, Missouri (gray squares). Both the population data and mean annual river discharge are presented as 10-year moving averages. The black and white diamond corresponds to the year the impoundment caused by R.L. Harris Dam (i.e., Lake Harris) was filled. The vertical reference line denotes an important regulatory milestone. C) A NMDS of mean species abundances by reach as derived from electrofishing grid efforts conducted annually in the Tallapoosa River from 2005–2013. D) The NMDS of mean species abundances by reach coded by time period (2005–2009 and 2010–2013).

The ordination of the data collected from the Tallapoosa River also revealed groupings related to the reaches evaluated (Fig 6). The ANOSIM results indicated, as with the other rivers, that there were significant differences in fish assemblages among reaches (*p* = 0.001; *Global R* = 0.498). Pairwise comparisons also suggested significant differences in fish assemblages between the majority of reaches (*p* = 0.001) with the exception of the comparison between the Wadley and Lower reaches (*p* = 0.1). The average dissimilarity between reaches ranged from a low of 36.0 for the comparison of the Wadley and Lower reaches to a maximum of 58.2 for the comparison of the Upper and the Malone reaches. The SIMPER results suggest that the assemblage in the Upper reach is not like the reaches below R.L. Harris Dam. The bullhead minnow *Pimephales vigilax* contributed the largest percent to the dissimilarities between reaches (Upper versus Malone, 12.2%; Upper versus Wadley, 14.6%; Upper versus Lower, 13.2%). Other fish species contributing ≥ 10% to the dissimilarities between the upper reach and the lower reaches include the Alabama shiner *Cyprinella callistia*, speckled darter *Etheostoma stigmaeum*, and the largescale stoneroller *Campostoma oligolepis*. For the reaches below R.L. Harris Dam, differences in abundances of the Alabama shiner contributed the largest percent to the dissimilarities for all pairwise comparisons (Malone versus Wadley, 18.8%; Malone versus Lower, 19.9%, Wadley versus Lower, 14.9%). Abundances of Alabama shiner were lowest in the Malone reach when compared to all other reaches; including the Upper reach. Differences in the abundances of the bronze darter *Percina palmaris* and the lipstick darter *Etheostoma chuckwachatte* contributed > 10% to the dissimilarity of all three pairwise comparisons of the reaches below R.L. Harris Dam. The grouping of data into time periods did not result in clear groupings in the ordination (Fig 6D). The lack of a strong temporal component was further confirmed by the ANOSIM for the three reaches below the R.L. Harris Dam (Malone: *p* = 0.33, Global R = 0.069; Wadley: *p* = 0.39, *Global R* = 0.038; Lower: *p* = 0.113, *Global R* = 0.21). However, there was a significant difference between the two time periods for the Upper reach (*p* = 0.008, *Global R* = 0.42). The SIMPER analyses comparing the two time periods for the Upper reach suggest that the dissimilarity between time periods (average dissimilarity = 35.4) was due to decreases in the abundance of several fish species with the largescale stoneroller, bullhead minnow, Alabama hogsucker *Hypentelium etowanum*, and speckled darter all contributing > 8% to the dissimilarity.

**Fig 7:**
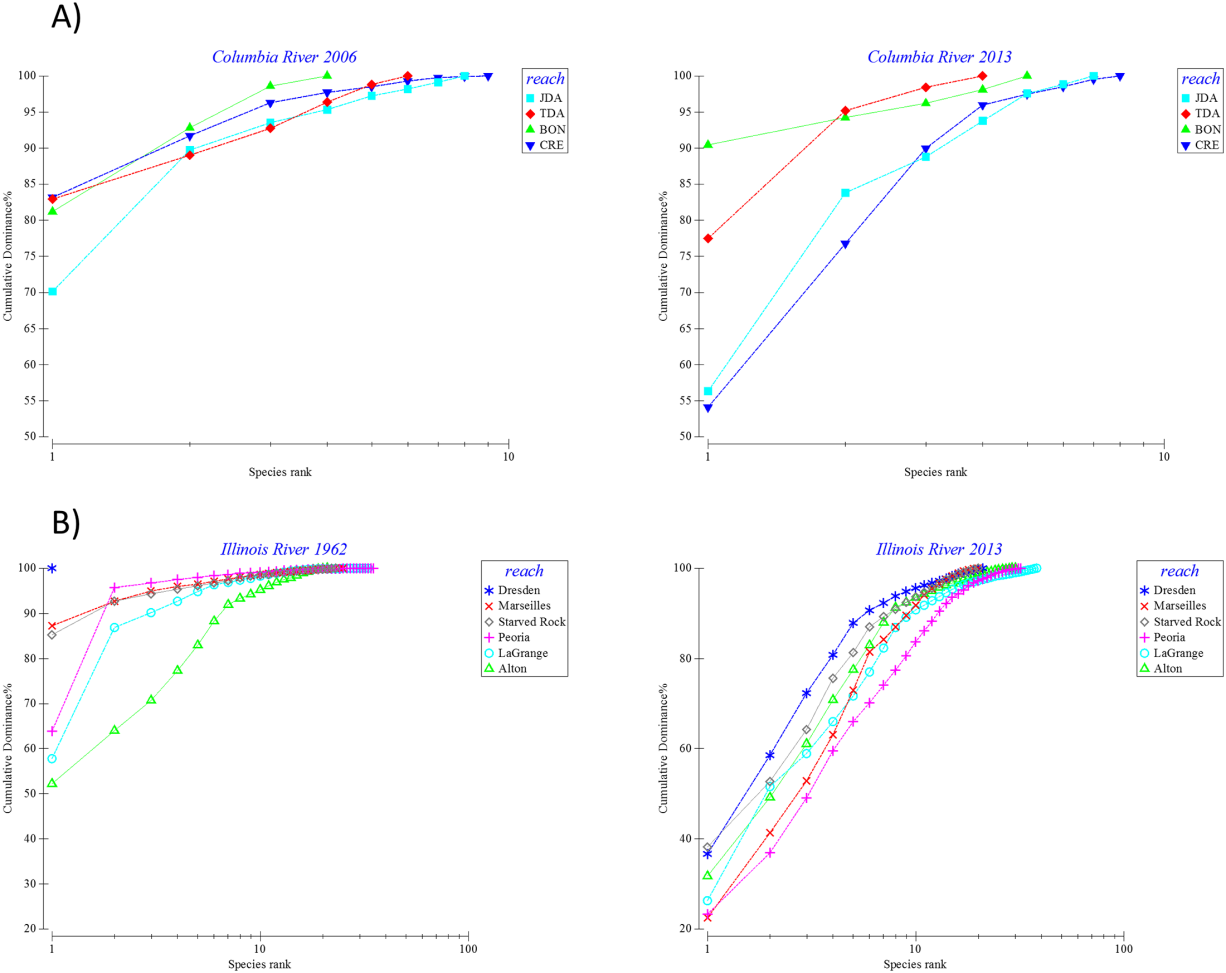
K-dominance plots for native fish assemblages in the A) Columbia and B) Illinois Rivers derived from fish monitoring programs in each of these river systems. Data are mean native species abundances summarized by reach and for two time periods for each river.

**Fig 8:**
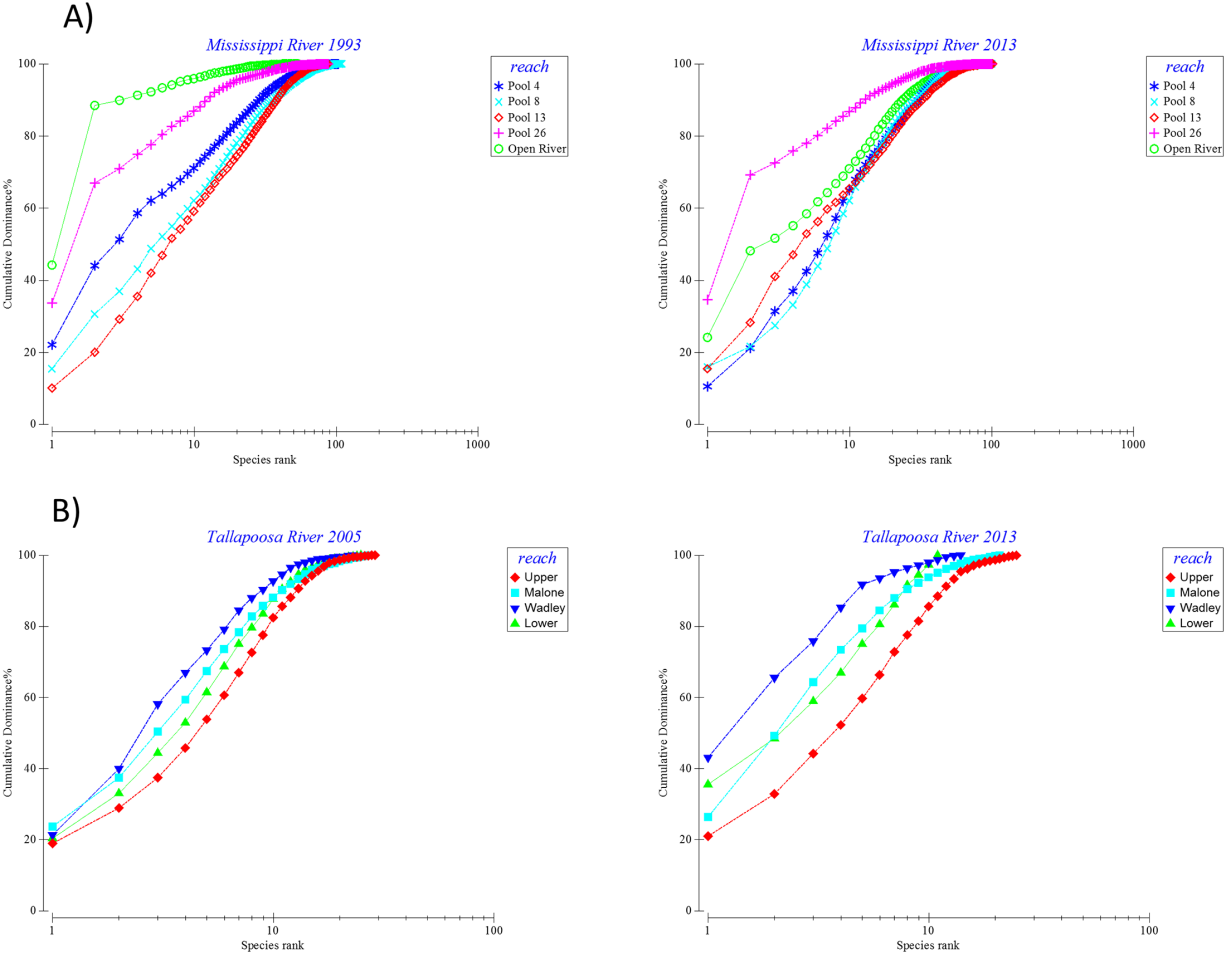
K-dominance plots for native fish assemblages in the A) Mississippi and B) Tallapoosa Rivers derived from fish monitoring programs in each of these river systems. Data are mean native species abundances summarized by reach and for two time periods for each river.

The k-dominance plots suggest that there were spatial and temporal trends in native species evenness and richness and that the trends were generally more evident in rivers with more native species and for programs that have been in place longer. Since the number and abundance of native species in the Colorado River were very low, k-dominance plots were not done. For the Columbia River, the dominance curves show a slight trend towards decreasing native species richness in Bonneville and The Dalles reaches and suggest that native species evenness increased from 2006 to 2013 in three of the four reaches (Fig 7A). However, the estimated native species richness in the Columbia River, based on the gears used and habitats sampled, was low compared to the other rivers evaluated. The dominance plots for the Illinois River, the river with the longest time series, suggest marked differences in species evenness from 1962 to 2013 for all of the reaches (Fig 7B). No consistent spatial trends in native species evenness were evident across time periods but there was a suggestion that native species evenness was highest in the Alton reach, furthest downstream, in the early years but that by 2013 native species evenness was highest in the Peoria reach and lowest in Dresden and Starved Rock. The dominance plots for the Illinois River also suggest that native species richness was generally greater for the three downriver reaches (i.e., Peoria, LaGrange, and Alton) versus the three upriver reaches (i.e., Dresden, Marseilles, and Starved Rock). For the Mississippi River, Pools 4 and 8 had higher native species evenness than Pool 26 and Open River reaches during both 1993 and 2013 (Fig 8A). No clear spatial or temporal trends in native species richness were evident from the dominance plots for most reaches on the Mississippi River. However, improvements in species evenness were evident from 1993 to 2013 for Pool 4 and the Open River reach. For the Tallapoosa River, the dominance plots suggest that native species evenness and richness were similar among reaches during 2005. In 2013, however, native species evenness decreased markedly in the Wadley reach (Fig 8B). Native species richness was similar among reaches during 2005 but lower in the Wadley and Lower reaches than in the Upper Tallapoosa and Malone reaches during 2013.

## Discussion

Long-term monitoring programs in large rivers provide unique case studies that can help us understand the cumulative effects of management and stressors at spatial and temporal scales relevant to the effective management of complex river ecosystems. Large river ecosystems are heavily affected by the cumulative and potentially synergistic effects of multiple stressors [37] including catchment disturbance, pollution, and water resource development [38]. Understanding processes affecting patterns in large river natural resources in the context of landscape level stressors requires knowledge of trends at multiple spatial and temporal scales [38,39,40,41,42,43,44]. Poff et al. [43] suggest that our understanding of river ecosystems would be enhanced by considering spatial scales relevant to river management, promoting a greater emphasis on learning through case studies, and synthesizing data across studies. Using data from multiple monitoring efforts, we were able to document significant spatial and temporal trends in fish assemblages within several large rivers across a variety of freshwater ecoregions [45].

Understanding the nature and extent of current trends in fish assemblages is necessary to better understand the effects of management and landscape levels stressors on biodiversity and fisheries in large rivers. The trends in fish assemblages we observed, when viewed in the context of data describing stressors, should provide insight about the effects of stressors on fish assemblages in large rivers. The five rivers examined in this study have commonalities with respect to stressors, and to some extent management actions to mitigate the effects of stressors, that could affect fish assemblages. For instance, all of the river systems in this study have been affected by the construction and operation of dams (Figs 2–6). Dams, whether they are for flood control or navigation, have predictable consequences that affect habitat connectivity, seasonal discharge patterns (Fig 3B), water temperature (Fig 2B), and habitat and have been shown to significantly affect the ecology of riverine systems [46,47] including negatively affecting fish diversity [48]. Dams also affect the distributions of sediments, organic matter, and contaminants [40,49] that can affect the distribution of fishes in rivers [50,51,52,53].

Our results suggest that long-term monitoring data could be used to better understand how dams interact with reach level geomorphology to structure fish assemblages in large rivers. Since fish assemblage structure has been shown to be affected by flow regime [47], channel morphology and geomorphic factors [54,55], and substrate composition, all of which are altered by the presence of dams, the trends in fish assemblages we observed in each river should provide an opportunity to better understand the effects of dams on large river fish assemblages. The spatial and temporal trends we observed in the Colorado River suggest that the operation of Glen Canyon Dam, including actions to lessen the effects of the dam on ESA listed fish species (e.g., humpback chub *Gila cypha*), are affecting fish assemblages. Our results show a distinct gradient of fish assemblages from the tailwater of Glen Canyon Dam that was dominated by non-native rainbow trout to the Lower reach where the fish assemblage was more diverse and contained more native fishes. The gradient in fish assemblage structure suggests the dam has created a gradient of habitat conditions that benefit different components of the Colorado River fish assemblage. The operation and management of the Federal Columbia River Power System (FCRPS), in particular to address the requirements of the listing of Pacific salmon populations (*Onchorhynchus spp*.) under the Endangered Species Act (ESA), has also likely affected the spatial and temporal trends in fish assemblage structure we observed in the Columbia River. In general, the alteration of the Columbia River from a relatively high gradient system to a series of low velocity impoundments has created favorable conditions for very few native (e.g., northern pikeminnow *Ptychocheilus oregonensis*) and many non-native fishes (e.g., smallmouth bass *Micropterus dolomieu*, walleye, yellow perch) [56,57]. Our results indicate that non-native fishes were less abundant in the un-impounded estuary than in Bonneville, The Dalles, and John Day reaches suggesting that the effects of impoundment have benefited non-native fishes. In addition, the predictable consequences of impoundment [58] and restricted passage through Columbia River dams has resulted in functionally isolated populations of resident fish [59] that now depend on reach specific conditions to sustain production. The UMR is also controlled and regulated by 27 dams and other river engineering structures [60] that have altered the hydrologic, sediment transport, geomorphic, water-quality, and ecologic characteristics of the river [60]. Johnson and Hagerty [6] noted that navigation dams on the UMR (Fig 5) are designed to raise the year round depth of the reach and prevent summer low water events; both affect the availability, quality and access to important floodplain and in-channel fish habitats, particularly fish spawning habitats. Johnson and Haggerty [6] posited that the effects of navigation dams are likely the cause for temporal shifts in assemblage composition. Irwin and Freeman [27] described the reaches of the Tallapoosa River below R.L. Harris Dam as strongly discharge-regulated. Irwin and Freeman [27] suggest that power peaking discharges from R.L. Harris Dam are affecting the aquatic community, including fishes, in the Tallapoosa River. Prior to implementation of pulsing discharges in 2005, low discharges were lower in magnitude, more frequent, and discharge conditions were less stable [27]. When adaptive discharge management ensued in 2005 (Fig 6), hydraulic conditions improved; however, our results indicate that a fish assemblage level response has not.

Spatial and temporal patterns of land use may have also affected the trends in fish assemblages we observed. All of the rivers examined in this study have also been subjected to extensive land use development. The term “land use” can include a variety of activities that produce very different responses; agricultural development in the Midwest corn producing regions can produce sedimentation and nutrient issues whereas urbanization and associated practices such as channelization can affect the localized, downstream distribution of fishes in rivers due to changes in habitat and flow responses in addition to water quality impacts [49,61]. Moreover, stressors such as contaminant concentrations [62], sedimentation rates [63], and water quality [64] that affect fish assemblages produce effects at different spatial and temporal scales depending on hydroclimatology and land use activity. That we were able to document spatial and temporal trends in fish assemblages over the range of programs evaluated suggests that data from disparate monitoring programs could provide insight into the effects of land use on large river fish assemblages. Long term monitoring programs can also provide the opportunity to view the effects of land use through time and in the context of stressors such as climate change where having an extended temporal framework would be beneficial. For example, McClelland et al. [7] suggest that large habitat improvement projects throughout the Illinois River system have resulted in positive changes to the fish assemblage [65]. However, accelerating sediment deposition in critical backwater habitats may mask many of those gains. The UMR has also been affected by the cumulative influence of more than 75 large habitat rehabilitation and enhancement projects completed since the 1990’s. These projects include summer water level drawdowns, island re-construction, backwater and side channel dredging, and fish passage improvements [6]. Johnson and Hagerty [6] discussed trends in stressors that affect fish assemblages and suggest that substantial improvements in some conditions have occurred since the 1960s due to Clean Water Act (CWA) regulations and changes in land use practices.

The trends in fish assemblages we observed provide an opportunity to better understand how spatial and temporal trends in water quality and contaminants are affecting large river fishes. Trends in water quality are variable within and across the river systems evaluated in this study; some rivers exhibiting marked improvements in water quality while others do not. In river systems where improvements in water quality are noted, our results suggest positive changes in fish assemblages have occurred over time (e.g., the Illinois and Mississippi rivers); while in river systems where water quality conditions have not improved significantly (e.g., the Tallapoosa River) there is evidence to suggest negative effects (6,22,27). Spatial and temporal changes in Colorado River water temperatures caused by the construction and operation of Glen Canyon Dam and drought (Fig 2B) have differentially affected native and non-native fishes [31,66,67,68,69,70]. Similarly, the construction of the FCRPS has altered the water temperature regime in the Columbia River and affected native and non-native fishes in different ways [71]. In addition to changes in the water temperature regime, fish tissue, sediment, and water contaminant concentrations in the Columbia River have been shown to vary spatially [49,72]. Fish assemblages have been shown to be structured along gradients of water temperature and contaminant concentrations in other systems [73]. Both Pegg and McClelland [22] and McClelland et al. [7] suggest that CWA driven improvements in water quality have led to positive changes in fish assemblage structure in the Illinois River (Fig 7B). Improvements in water quality regulation and policy associated with the CWA have been cited in other river systems as being a driver of positive changes in fish assemblages. For example, Lohner and Dixon [74] suggest that improvements in Ohio River fish populations coincide with water quality improvements resulting from the CWA. However, in the UMR, the effects of the CWA on fish assemblages may be less clear because of the confounding effects of other factors affecting fish assemblages. Johnson and Hagerty [6] suggest that water quality has improved in the UMR because of the CWA but note that temporal trends in water quality parameters were variable across reaches and likely related to spatial differences in land use. Irwin and Freeman [27] noted that lowered temperatures resulting from pulsed hypolimnetic releases from the reach above R.L. Harris Dam on the Tallapoosa River likely delay spawning periods, impede hatching success, and decrease rates of larval development for native fishes. Lower temperatures from hypolimnetic releases have been shown to affect populations of native fishes in other systems [52,75,76].

Long term monitoring programs offer an opportunity to better understand the long term effects of established non-native fishes, and also the effects of newly introduced non-native fishes, on native fish assemblages. We demonstrated spatial and temporal trends in select non-native fishes in most of the rivers; the Tallapoosa River being the exception where few non-native fishes were documented. However, introductions of non-native fishes have clearly influenced fish assemblage structure in large rivers. For instance, because of the interactions of non-native rainbow trout with native fishes [70], the management of this fishery affects the trends we observed in the Colorado River. The rainbow trout fishery near Lees Ferry (Upper reach) is currently managed as a recreational fishery [77]; but mechanical removal of rainbow trout has occurred in the Middle reach near the confluence of the Little Colorado River as recently as 2009 to reduce negative interactions with humpback chub [68,70]. Similar to that observed for the Colorado River, the introduction of non-native fishes has clearly affected the native fish assemblage in the Columbia River [57]. Channel catfish, smallmouth bass, and walleye that have been introduced into the Columbia River have all been shown to prey upon or compete with native fish species [57,78]. For the Illinois and Mississippi rivers, the arrival of successive invasive species (e.g., the zebra mussel *Dreissena polymorpha* and bighead and silver carp) have the potential to erode gains made from improved water quality [16,25,79,80,81].

Despite the fact that rivers and streams harbor a diverse and unique array of species, habitats, and ecosystems, including some that are threatened and of great value to human society, biodiversity conservation has received relatively little attention compared to other aquatic (e.g., ocean) and terrestrial (e.g., tropical rain forests) systems [82]. The trends in native species diversity we observed, while affected by the different durations of the programs (from < 10 years to > 50 years), suggest an opportunity to better understand how common stressors that can vary spatially and temporally within and between river basins are affecting large river native fish biodiversity. As mentioned previously, the transformation of the Columbia River into a series of slow-moving low gradient impoundments has created habitats that non-native fishes, that compete and prey upon native fishes, thrive in, such that significant populations of non-native predators have become established that affect native fishes. Conversely, tailwaters below dams create areas of relatively high velocities where there may have been slower water velocities, as has happened in the Tallapoosa River. Travnichek and Maceina [83] also found that species richness and diversity of shallow water fishes in the Tallapoosa River were reduced below two hydroelectric dams compared with unmodified river segments and suggest that the reduction in species adapted to fluvial environments below dams on the Tallapoosa River was the cause. On the other hand, the relative effects of channelization that lead to altered sediment transport patterns [84], reduced floodplain connectivity [85,86], altered main channel width and depth, and restricted channel meandering [87,88,89] can also affect native fishes but the effects likely vary within and between basins because of differences in geographic settings and the relative effects of land uses such as urbanization and agricultural development. If conserving aquatic biodiversity in large rivers is desired, developing a better understanding of trends and factors causing the trends will be necessary to make decisions and formulate mitigation.

While having a dedicated regional or national standardized large river fish monitoring effort would better facilitate our understanding of continental trends in large river fisheries, we feel that information can and should be derived from existing efforts. Indeed, since large rivers are hierarchically structured with both local (e.g., dams, point-source pollution) and landscape-scale (e.g., climate patterns, land use patterns) drivers and stressors acting on fish assemblages simultaneously [90,91], we feel that much can be learned about various factors affecting fish assemblages and other large river resources at regional and continental scales despite the inherent differences in monitoring programs. Given the benefits provided by large rivers to local and regional economies, to a diverse array of commercial, cultural, and recreational constituents, and because of large river’s importance to the conservation of the continents biodiversity [92], deriving a better understanding of trends and stressors is imperative. By using information garnered from existing programs over broad geographical scales, we can also identify opportunities for learning across established programs [18] and provide information that will help those interested in beginning new programs to assess the status and trends of natural resources in large rivers. Increased knowledge of factors affecting large river resources at broad geographical scales will help managers better formulate policy that addresses emerging issues at spatial scales much larger than the individual programs allow.

## Supporting information

S1 Table Common name, scientific name, river where species was present, and whether the fish species is native to the river (Y = yes; N = No) for fishes captured as part of monitoring efforts in the Colorado, Columbia, Illinois, Mississippi, and Tallapoosa rivers. (PDF)
