# Supplementary material for: Can data from disparate long-term fish monitoring programs be used to increase our understanding of regional and continental trends in large river assemblages?
Source: PLoS One. 2018 Jan 24;13(1):e0191472. doi: 10.1371/journal.pone.0191472 (PMC5783367; doi:10.1371/journal.pone.0191472)
Supplement: S1 Table — (PDF) [file pone.0191472.s001.pdf]

S1 Table. Common name, scientific name, river where species was present, and whether the fish species is native to the river (Y = yes; N = No) for fishes captured as part of monitoring efforts in the Colorado, Columbia, Illinois, Mississippi, and Tallapoosa rivers.

Common Name	Scientific Name	Rivers where species present	Native to Colorado	Native to Columbia	Native to Illinois	Native to Mississippi	Native to Tallapoosa
Alabama Bass	Micropterus henshalli	Tallapoosa					Y
Alabama Hog Sucker	Hypentelium etowanum	Tallapoosa					Y
Alabama Shiner	Cyprinella callistia	Tallapoosa					Y
American Brook Lamprey	Lethenteron appendix	Mississippi				Y	
American Eel	Anguilla rostrata	Illinois, Mississippi			Y	Y	
American Shad	Alosa sapidissima	Columbia		N			
Banded Darter	Etheostoma zonale	Mississippi				Y	
Banded Killifish	Fundulus diaphanus	Illinois			Y		
Bigeye Chub	Notropis amblops	Mississippi				Y	
Bigeye Shiner	Notropis boops	Mississippi				Y	
Bighead Carp	Hypopthalmichthys nobilis	Illinois, Mississippi			N	N	
Bigmouth Buffalo	Ictiobus cyprinellus	Illinois, Mississippi			Y	Y	
Bigmouth Shiner	Notropis dorsalis	Illinois, Mississippi			Y	Y	
Black Buffalo	Ictiobus niger	Illinois, Mississippi			Y	Y	
Black Bullhead	Ameiurus melas	Colorado, Illinois, Mississippi	N		Y	Y	
Black Crappie	Pomoxis nigromaculatus	Colorado, Illinois, Mississippi, Tallapoosa	N		Y	Y	Y
Black Madtom	Noturus funebris	Tallapoosa					Y
Black Redhorse	Moxostoma duqesnei	Illinois, Tallapoosa			Y		Y

S1 Table (continued). Common name, scientific name, river where species was present, and whether the fish species is native to the river (Y = yes; N = No) for fishes captured as part of monitoring efforts in the Colorado, Columbia, Illinois, Mississippi, and Tallapoosa rivers.

Common Name	Scientific Name	Rivers where species present	Native to Colorado	Native to Columbia	Native to Illinois	Native to Mississippi	Native to Tallapoosa
Blacknose Dace	Rhinichthys atratulus	Illinois, Mississippi			Y	Y	
Blackside Darter	Percina maculata	Illinois, Mississippi			Y	Y	
Blackspotted Topminnow	Fundulus olivaceus	Mississippi, Tallapoosa				Y	Y
Blackstripe Topminnow	Fundulus notatus	Illinois, Mississippi			Y	Y	
Blacktail Redhorse	Moxostoma poecilurum	Tallapoosa					Y
Blacktail Shiner	Cyprinella venusta	Mississippi, Tallapoosa				Y	Y
Bleeding Shiner	Luxilus zonatus	Mississippi				Y	
Blue Catfish	Ictalurus furcatus	Illinois, Mississippi			Y	Y	
Blue Sucker	Cycleptus elongatus	Mississippi				Y	
Bluegill	Lepomis macrochirus	Colorado, Columbia, Illinois, Mississippi, Tallapoosa	N	N	Y	Y	Y
Bluehead Chub	Nocomis leptocephalus	Tallapoosa					Y
Bluehead Sucker	Catostomus discobolus	Colorado	Y				
Bluntnose Darter	Etheostoma chlorosomum	Illinois, Mississippi			Y	Y	
Bluntnose Minnow	Pimephales notatus	Illinois, Mississippi			Y	Y	
Bowfin	Amia calva	Illinois, Mississippi			Y	Y	
Brassy Minnow	Hybognathus hankinsoni	Mississippi				Y	

S1 Table (continued). Common name, scientific name, river where species was present, and whether the fish species is native to the river (Y = yes; N = No) for fishes captured as part of monitoring efforts in the Colorado, Columbia, Illinois, Mississippi, and Tallapoosa rivers.

**Common Name**	**Scientific Name**	**Rivers where species present**	**Native to Colorado**	**Native to Columbia**	**Native to Illinois**	**Native to Mississippi**	**Native to Tallapoosa**
Bridgelip Sucker	Catostomus columbianus	Columbia		Y			
Bronze Darter	Percina palmaris	Tallapoosa					Y
Brook Silverside	Labidesthes sicculus	Illinois, Mississippi			Y	Y	
Brook Stickleback	Culaea inconstans	Mississippi				Y	
Brook Trout	Salvelinus fontinalis	Colorado	N				
Brown Bullhead	Ameiurus nebulosus	Illinois, Mississippi			Y	Y	
Brown Trout	Salmo trutta	Colorado, Mississippi	N			N	
Bullhead Minnow	Pimephales vigilax	Illinois, Mississippi, Tallapoosa			Y	Y	Y
Burbot	Lota lota	Mississippi				Y	
Burrhead Shiner	Notropis asperifrons	Tallapoosa					Y
Central Mudminnow	Umbra limi	Mississippi				Y	
Central Stoneroller	Campostoma anomalum	Illinois, Mississippi			Y	Y	
Channel Catfish	Ictalurus punctatus	Colorado, Columbia, Illinois, Mississippi, Tallapoosa	N	N	Y	Y	Y
Channel Shiner	Notropis wickliffi	Mississippi				Y	
Chestnut Lamprey	Ichthyomyzon castaneus	Mississippi				Y	
Chinook Salmon	Oncorhynchus tshawytscha	Columbia		Y			

S1 Table (continued). Common name, scientific name, river where species was present, and whether the fish species is native to the river (Y = yes; N = No) for fishes captured as part of monitoring efforts in the Colorado, Columbia, Illinois, Mississippi, and Tallapoosa rivers.

Common Name	Scientific Name	Rivers where species present	Native to Colorado	Native to Columbia	Native to Illinois	Native to Mississippi	Native to Tallapoosa
Chiselmouth Chub	Acrocheilus alutaceus	Columbia		Y			
Chum Salmon	Oncorhynchus keta	Columbia		Y			
Coho Salmon	Oncorhynchus kisutch	Columbia		Y			
Common Carp	Cyprinus carpio	Colorado, Columbia, Illinois, Mississippi	N	N	N	N	
Common Shiner	Luxilus cornutus	Illinois, Mississippi			Y	Y	
Creek Chub	Semotilus atromaculatus	Illinois, Mississippi, Tallapoosa			Y	Y	Y
Creek Chubsucker	Erimyzon oblongus	Mississippi				Y	
Crystal Darter	Ammocrypta asprella	Mississippi				Y	
Cutthroat Trout	Oncorhynchus clarkii	Colorado, Columbia	N	Y			
Dusky Darter	Percina sciera	Mississippi				Y	
Emerald Shiner	Notropis atherinoides	Illinois, Mississippi			Y	Y	
Eulachon	Thaleichthys pacificus	Columbia		Y			
Fantail Darter	Etheostoma flabellare	Mississippi				Y	
Fathead Minnow	Pimephales promelas	Colorado, Illinois, Mississippi	N		Y	Y	
Flannelmouth Sucker	Catostomus latipinnis	Colorado	Y				
Flathead Catfish	Pylodictis olivaris	Illinois, Mississippi, Tallapoosa			Y	Y	Y

S1 Table (continued). Common name, scientific name, river where species was present, and whether the fish species is native to the river (Y = yes; N = No) for fishes captured as part of monitoring efforts in the Colorado, Columbia, Illinois, Mississippi, and Tallapoosa rivers.

Common Name	Scientific Name	Rivers where species present	Native to Colorado	Native to Columbia	Native to Illinois	Native to Mississippi	Native to Tallapoosa
Flier	Centrarchus macropterus	Mississippi				Y	
Freckled Madtom	Noturus nocturnus	Illinois, Mississippi			Y	Y	
Freshwater Drum	Aplodinotus grunniens	Illinois, Mississippi			Y	Y	
Ghost Shiner	Notropis buchanani	Illinois, Mississippi			Y	Y	
Gizzard Shad	Dorosoma cepedianum	Colorado, Illinois, Mississippi, Tallapoosa	N		Y	Y	Y
Golden Redhorse	Moxostoma erythrurum	Illinois, Mississippi, Tallapoosa			Y	Y	Y
Golden Shiner	Notemigonus crysoleucas	Colorado, Illinois, Mississippi, Tallapoosa	N		Y	Y	Y
Goldeye	Hiodon alosoides	Illinois, Mississippi			Y	Y	
Goldfish	Carassius auratus	Columbia, Illinois, Mississippi		N	N	N	
Grass Carp	Ctenopharyngodon Idella	Illinois, Mississippi			N	N	
Grass Pickerel	Esox americanus vermiculatus	Illinois, Mississippi			Y	Y	
Green Sturgeon	Acipenser medirostris	Columbia		Y			
Green Sunfish	Lepomis cyanellus	Colorado, Illinois, Mississippi, Tallapoosa	N		Y	Y	Y
Greenside Darter	Etheostoma blennioides	Mississippi				Y	
Highfin Carpsucker	Carpiodes velifer	Illinois, Mississippi			Y	Y	
Hornyhead Chub	Nocomis biguttatus	Illinois, Mississippi			Y	Y	
Humpback Chub	Gila cypha	Colorado	Y				

S1 Table (continued). Common name, scientific name, river where species was present, and whether the fish species is native to the river (Y = yes; N = No) for fishes captured as part of monitoring efforts in the Colorado, Columbia, Illinois, Mississippi, and Tallapoosa rivers.

**Common Name**	**Scientific Name**	**Rivers where species present**	**Native to Colorado**	**Native to Columbia**	**Native to Illinois**	**Native to Mississippi**	**Native to Tallapoosa**
Inland Silverside	Menidia beryllina	Mississippi				Y	
Iowa Darter	Etheostoma exile	Mississippi				Y	
Johnny Darter	Etheostoma nigrum	Illinois, Mississippi			Y	Y	
Lake Sturgeon	Acipenser fulvescens	Mississippi				Y	
Largemouth Bass	Micropterus salmoides	Colorado, Columbia, Illinois, Mississippi, Tallapoosa	N	N	Y	Y	Y
Largescale Stoneroller	Campostoma oligolepis	Mississippi, Tallapoosa				Y	Y
Largescale Sucker	Catostomun macroheilus	Columbia		Y			
Least Brook Lamprey	Lampetra aepyptera	Mississippi				Y	
Lined Chub	Hybopsis lineapunctata	Tallapoosa					Y
Lipstick Darter	Etheostoma chuckwachatte	Tallapoosa					Y
Logperch	Percina caprodes	Illinois, Mississippi			Y	Y	
Longear Sunfish	Lepomis megalotis	Illinois, Mississippi, Tallapoosa			Y	Y	Y
Longfin Smelt	Spirinchus thaleichthys	Columbia		Y			
Longnose Gar	Lepisosteus osseus	Illinois, Mississippi			Y	Y	
Mimic Shiner	Notropis volucellus	Mississippi				Y	
Mississippi Silvery Minnow	Hybognathus nuchalis	Illinois, Mississippi			Y	Y	
Mobile Logperch	Percina kathae	Tallapoosa					Y
Mooneye	Hiodon tergisus	Illinois, Mississippi			Y	Y	

S1 Table (continued). Common name, scientific name, river where species was present, and whether the fish species is native to the river (Y = yes; N = No) for fishes captured as part of monitoring efforts in the Colorado, Columbia, Illinois, Mississippi, and Tallapoosa rivers.

Common Name	Scientific Name	Rivers where species present	Native to Colorado	Native to Columbia	Native to Illinois	Native to Mississippi	Native to Tallapoosa
Mosquito Fish	Gambusia affinis	Colorado	N				
Mountain Whitefish	Prosopium williamsoni	Columbia		Y			
Mud Darter	Etheostoma asprigene	Illinois, Mississippi			Y	Y	
Muscadine Darter	Percina smithvanizi	Tallapoosa					Y
Muskellunge	Esox masquinongy	Mississippi				Y	
Northern Hogsucker	Hypentelium nigricans	Illinois, Mississippi			Y	Y	
Northern Pike	Esox lucius	Colorado, Illinois, Mississippi	N		Y	Y	
Northern Pikeminnow	Ptychocheilus oregonensis	Columbia		Y			
Northern Studfish	Fundulus catenatus	Mississippi				Y	
Orangespotted Sunfish	Lepomis humilis	Illinois, Mississippi			Y	Y	
Orangethroat Darter	Etheostoma spectabile	Mississippi				Y	
Ozark Minnow	Notropis nubilus	Mississippi				Y	
Pacific Lamprey	Entosphenus tridentatus	Columbia		Y			
Paddlefish	Polyodon spathula	Mississippi				Y	
Pallid Shiner	Notropis amnis	Mississippi				Y	
Peamouth Chub	Mylocheilus caurinus	Columbia		Y			
Pirate Perch	Aphredoderus sayanus	Mississippi				Y	
Plains Killifish	Zebrinus fundulus	Colorado	N				

S1 Table (continued). Common name, scientific name, river where species was present, and whether the fish species is native to the river (Y = yes; N = No) for fishes captured as part of monitoring efforts in the Colorado, Columbia, Illinois, Mississippi, and Tallapoosa rivers.

Common Name	Scientific Name	Rivers where species present	Native to Colorado	Native to Columbia	Native to Illinois	Native to Mississippi	Native to Tallapoosa
Plains Minnow	Hybognathus placitus	Mississippi				Y	
Pretty Shiner	Lythrurus bellus	Tallapoosa					Y
Prickly Sculpin	Cottus asper	Columbia		Y			
Pugnose Minnow	Opsopoeodus emiliae	Illinois, Mississippi			Y	Y	
Pumpkinseed	Lepomis gibbosus	Columbia, Illinois, Mississippi		N	Y	Y	
Quillback	Carpiodes cyprinus	Illinois, Mississippi			Y	Y	
Rainbow Smelt	Osmerus mordax	Mississippi				N	
Rainbow Trout	Oncorhynchus mykiss	Colorado, Illinois	N		Y		
Razorback Sucker	Xyrauchen texanus	Colorado	Y				
Red Shiner	Cyprinella lutrensis	Colorado, Illinois, Mississippi	N		Y	Y	
Redbreast Sunfish	Lepomis auritus	Tallapoosa					Y
Redear Sunfish	Lepomis microlophus	Illinois, Mississippi, Tallapoosa			Y	Y	Y
Redeye Bass	Micropterus tallapoosae	Tallapoosa					Y
Redfin Shiner	Notropis umbratilis	Illinois			Y		
Redside Shiner	Richardsonius balteatus	Colorado	N				
Redspotted Sunfish	Lepomis miniatus	Mississippi				Y	
Ribbon Shiner	Notropis fumeus	Illinois			Y		
Riffle Minnow	Phenacobius catostomus	Tallapoosa					Y

S1 Table (continued). Common name, scientific name, river where species was present, and whether the fish species is native to the river (Y = yes; N = No) for fishes captured as part of monitoring efforts in the Colorado, Columbia, Illinois, Mississippi, and Tallapoosa rivers.

**Common Name**	**Scientific Name**	**Rivers where species present**	**Native to Colorado**	**Native to Columbia**	**Native to Illinois**	**Native to Mississippi**	**Native to Tallapoosa**
River Carpsucker	Carpiodes carpio	Illinois, Mississippi			Y	Y	
River Chub	Nocomis micropogon	Mississippi				Y	
River Darter	Percina shumardi	Mississippi				Y	
River Redhorse	Moxostoma carinatum	Illinois, Mississippi			Y	Y	
River Shiner	Notropis blennius	Illinois, Mississippi			Y	Y	
Rock Bass	Ambloplites rupestris	Illinois, Mississippi			Y	Y	
Rough Shiner	Notropis baileyi	Tallapoosa					Y
Round Goby	Neogobius melanostomus	Illinois, Mississippi			N	Y	
Rudd	Scardinius erythrophthalmus	Mississippi				N	
Sand Shiner	Notropis stramineus	Colorado, Illinois, Mississippi	N		Y	Y	
Sauger	Stizostedion canadense	Illinois, Mississippi			Y	Y	
Shadow Bass	Ambloplites ariommus	Tallapoosa					Y
Shiner	Cyprinella lutrensis	Colorado	N				
Shoal Chub	Macrhybopsis aestivalis	Tallapoosa					Y
Shorthead Redhorse	Moxostoma macrolepidotum	Illinois, Mississippi			Y	Y	
Shortnose Gar	Lepisosteus platostomus	Illinois, Mississippi			Y	Y	
Shovelnose Sturgeon	Scaphirhynchus platorynchus	Mississippi				Y	

S1 Table (continued). Common name, scientific name, river where species was present, and whether the fish species is native to the river (Y = yes; N = No) for fishes captured as part of monitoring efforts in the Colorado, Columbia, Illinois, Mississippi, and Tallapoosa rivers.

Common Name	Scientific Name	Rivers where species present	Native to Colorado	Native to Columbia	Native to Illinois	Native to Mississippi	Native to Tallapoosa
Sicklefin Chub	Macrhybopsis meeki	Mississippi				Y	
Silver Carp	Hypopthalmichthys molitrix	Illinois, Mississippi			N	N	
Silver Chub	Macrhybopsis storeriana	Illinois, Mississippi			Y	Y	
Silver Lamprey	Ichthyomyzon unicuspis	Mississippi				Y	
Silver Redhorse	Moxostoma anisurum	Illinois, Mississippi			Y	Y	
Silverband Shiner	Notropis shumardi	Illinois, Mississippi			Y	Y	
Silverjaw Minnow	Ericymba buccata	Illinois			Y		
Silverstripe Shiner	Notropis stilbius	Tallapoosa					Y
Skipjack Herring	Alosa chrysochloris	Illinois, Mississippi			Y	Y	
Slenderhead Darter	Percina phoxocephala	Illinois, Mississippi			Y	Y	
Slough Darter	Etheostoma gracile	Mississippi				Y	
Smallmouth Bass	Micropterus dolomieu	Colorado, Columbia, Illinois, Mississippi	N	N	Y	Y	
Smallmouth Buffalo	Ictiobus bubalus	Illinois, Mississippi			Y	Y	
Southern Redbelly Dace	Phoxinus erythrogaster	Illinois, Mississippi			Y	Y	

S1 Table (continued). Common name, scientific name, river where species was present, and whether the fish species is native to the river (Y = yes; N = No) for fishes captured as part of monitoring efforts in the Colorado, Columbia, Illinois, Mississippi, and Tallapoosa rivers.

Common Name	Scientific Name	Rivers where species present	Native to Colorado	Native to Columbia	Native to Illinois	Native to Mississippi	Native to Tallapoosa
Speckled Chub	Macrhybopsis aestivalis	Mississippi				Y	
Speckled Dace	Rhinichthys osculus	Colorado	Y				
Speckled Darter	Etheostoma stigmaeum	Tallapoosa					Y
Speckled Madtom	Noturus leptacanthus	Tallapoosa					Y
Spotfin Shiner	Cyprinella spiloptera	Illinois, Mississippi			Y	Y	
Spottail Shiner	Notropis hudsonius	Illinois, Mississippi			Y	Y	
Spotted Bass	Micropterus punctulatus	Illinois, Mississippi			Y	Y	
Spotted Gar	Lepisosteus oculatus	Illinois, Mississippi			Y	Y	
Spotted Sucker	Minytrema melanops	Mississippi, Tallapoosa				Y	Y
Spotted Sunfish	Lepomis punctatus	Illinois			Y		
Starhead Topminnow	Fundulus dispar	Mississippi				Y	
Starry Flounder	Platichthys dtellatus	Columbia		Y			
Steelcolor Shiner	Notropis whipplei	Illinois			Y		
Steelhead	Oncorhynchus mykiss	Columbia		Y			
Stippled Studfish	Fundulus bifax	Tallapoosa					Y
Stonecat	Noturus flavus	Mississippi				Y	
Striped Bass	Morone saxatilis	Colorado, Illinois, Mississippi	N		Y	N	

S1 Table (continued). Common name, scientific name, river where species was present, and whether the fish species is native to the river (Y = yes; N = No) for fishes captured as part of monitoring efforts in the Colorado, Columbia, Illinois, Mississippi, and Tallapoosa rivers.

Common Name	Scientific Name	Rivers where species present	Native to Colorado	Native to Columbia	Native to Illinois	Native to Mississippi	Native to Tallapoosa
Striped Mullet	Mugil cephalus	Mississippi				Y	
Striped Shiner	Luxilus chrysocephalus,	Illinois, Mississippi, Tallapoosa			Y	Y	Y
Sturgeon Chub	Macrhybopsis gelida	Mississippi				Y	
Suckermouth Minnow	Phenacobius mirabilis	Illinois, Mississippi			Y	Y	
Tadpole Madtom	Noturus gyrinus	Illinois, Mississippi			Y	Y	
Tallapoosa Darter	Etheostoma tallapoosae	Tallapoosa					Y
Tallapoosa Sculpin	Cottus tallapoosae	Tallapoosa					Y
Tallapoosa Shiner	Cyprinella gibbsi	Tallapoosa					Y
Threadfin Shad	Dorosoma petenense	Colorado, Illinois, Mississippi, Tallapoosa	N		Y	Y	Y
Threespine Stickleback	Gasterosteus aculeatus	Columbia		Y			
Trout Perch	Percopsis omiscomaycus	Illinois, Mississippi			Y	Y	
Utah Chub	Gila atraria	Colorado	N				
Utah Sucker	Catostomus ardens	Colorado	N				
Walleye	Stizostedion vitreum	Colorado, Columbia, Illinois, Mississippi	N	N	Y	Y	
Warmouth	Lepomis gulosus	Illinois, Mississippi, Tallapoosa			Y	Y	Y
Wedgespot Shiner	Notropis greenei	Mississippi				Y	
Weed Shiner	Notropis texanus	Mississippi, Tallapoosa				Y	Y

S1 Table (continued). Common name, scientific name, river where species was present, and whether the fish species is native to the river (Y = yes; N = No) for fishes captured as part of monitoring efforts in the Colorado, Columbia, Illinois, Mississippi, and Tallapoosa rivers.

Common Name	Scientific Name	Rivers where species present	Native to Colorado	Native to Columbia	Native to Illinois	Native to Mississippi	Native to Tallapoosa
Western Mosquitofish	Gambusia affinis	Illinois, Mississippi, Tallapoosa			Y	Y	Y
Western Sand Darter	Ammocrypta clara	Mississippi				Y	
Western Silvery Minnow	Hybognathus argyritis	Mississippi				Y	
White Bass	Morone chrysops	Illinois, Mississippi			Y	Y	
White Catfish	Ameiurus catus	Illinois			Y		
White Crappie	Pomoxis annularis	Columbia, Illinois, Mississippi		N	Y	Y	
White Perch	Morone americana	Illinois, Mississippi			N	N	
White Sturgeon	Acipenser transmontanus	Columbia		Y			
White Sucker	Catostomus commersoni	Illinois, Mississippi			Y	Y	
Yellow Bass	Morone mississippiensis	Illinois, Mississippi			Y	Y	
Yellow Bullhead	Ameiurus natalis	Colorado, Columbia, Illinois, Mississippi, Tallapoosa	N	N	Y	Y	Y
Yellow Perch	Perca flavescens	Colorado, Columbia, Illinois, Mississippi, Tallapoosa	N	N	Y	Y	N
